# Supplementary material for: Genomic analysis of an ultrasmall freshwater green alga, Medakamo hakoo
Source: Commun Biol. 2023 Jan 23;6:89. doi: 10.1038/s42003-022-04367-9 (PMC9871001; doi:10.1038/s42003-022-04367-9)
Supplement: Supplementary file 2 — Supplementary Information [file 42003_2022_4367_MOESM2_ESM.pdf]

Supplementary Information

Genomic analysis of an ultrasmall freshwater green alga, *Medakamo hakoo*

Shoichi Kato, Osami Misumi, Shinichiro Maruyama, Hisayoshi Nozaki, Yayoi Tsujimoto-Inui, Mari Takusagawa, Shigekatsu Suzuki, Keiko Kuwata, Saki Noda, Nanami Ito, Yoji Okabe, Takuya Sakamoto, Fumi Yagisawa, Tomoko M. Matsunaga, Yoshikatsu Matsubayashi, Haruyo Yamaguchi, Masanobu Kawachi, Haruko Kuroiwa, Tsuneyoshi Kuroiwa & Sachihiro Matsunaga

Communications Biology | ISSN 2399-3642 (online)

January, 2023

https://doi.org /10.1038/s42003-022-04367-9

Supplementary Figures

Supplementary Figure 1……………………………………………………………2

Supplementary Figure 2……………………………………………………………3

Supplementary Figure 3……………………………………………………………4

Supplementary Figure 4……………………………………………………………5

Supplementary Figure 5……………………………………………………………6

Supplementary Figure 6……………………………………………………………7

Supplementary Figure 7……………………………………………………………8

Supplementary Figure 8……………………………………………………………9-11

Supplementary Tables

Supplementary Table 1……………………………………………………………12

Supplementary Table 2……………………………………………………………13

Supplementary Table 3……………………………………………………………14

Supplementary Table 4……………………………………………………………15

Supplementary Table 5……………………………………………………………16

Supplementary Table 6……………………………………………………………17

Supplementary Table 7……………………………………………………………18

Supplementary Table 8……………………………………………………………19

Supplementary Note

Supplementary Note 1……………………………………………………………..20-21

Supplementary Data Contents…………………………………………………...22

Supplementary References…………………………………………………………22

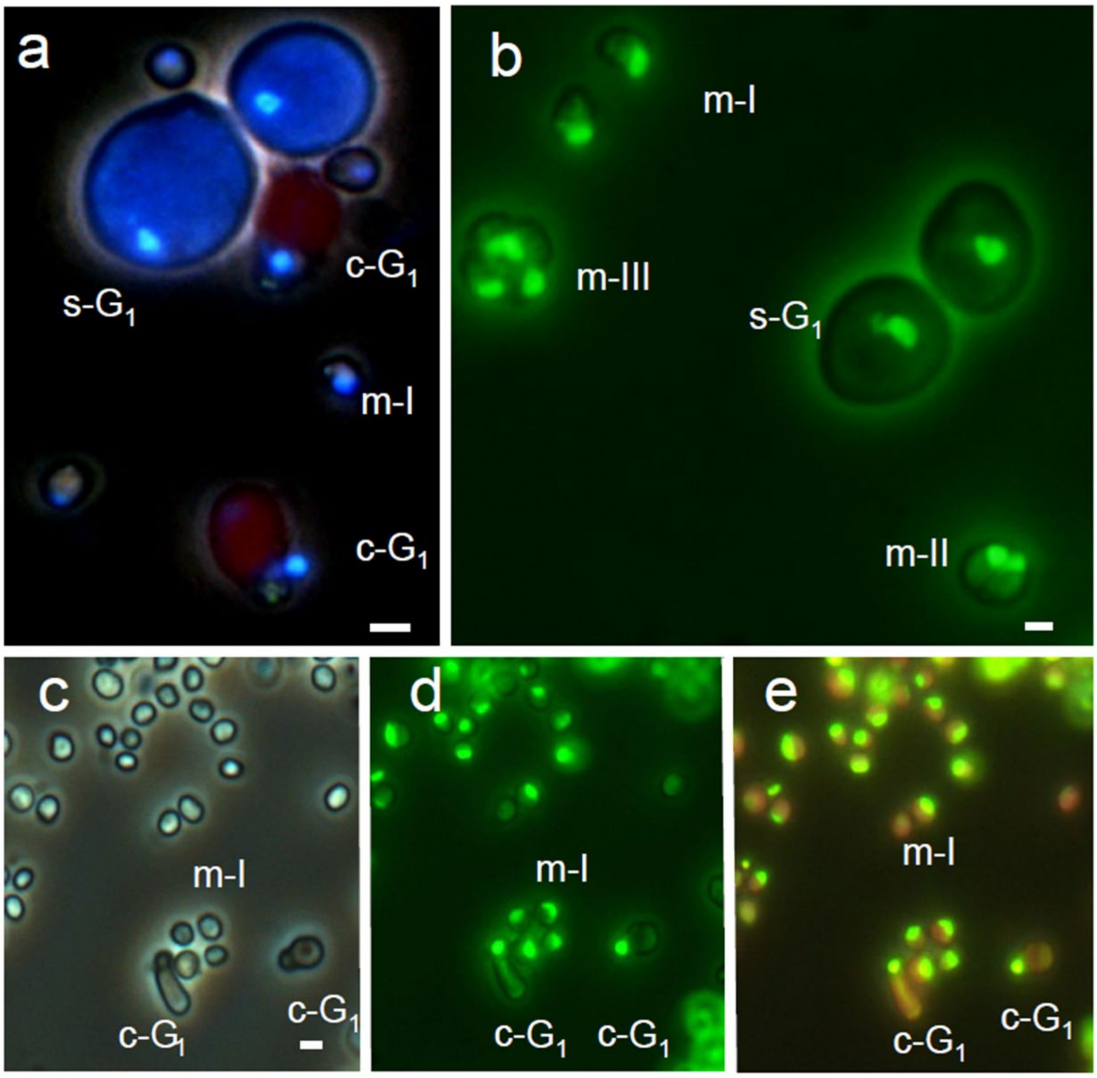

Supplementary Figure 1 Additional fluorescence images of *M. hakoo*, *C. merolae*, and S. cerevisiae. a Fluorescence image after the DAPI staining. The *S. cerevisiae* cells (s-G1), two *C. merolae* G1 phase cells (c-G1), and a few *M. hakoo* interphase cells (m-I) are presented. b Fluorescence image after the SYBR Green staining. The *S. cerevisiae* G1 phase cells (s-G1) and *M. hakoo* cells are presented. The *M. hakoo* cells are presented in the following phases: interphase (m-I), G1 phase right after two cell divisions (m-II), and G1 phase right after the cell division that results in the tetrad cell state (m-III). c–e Images of *C. merolae* G1 phase cells (c-G1) and *M. hakoo* cells (m-I). Phase-contrast image (c), green fluorescence image merged with the phase-contrast image (d), and the merged image combined with an autofluorescence image (e) are presented. Bars represent 1 μm.

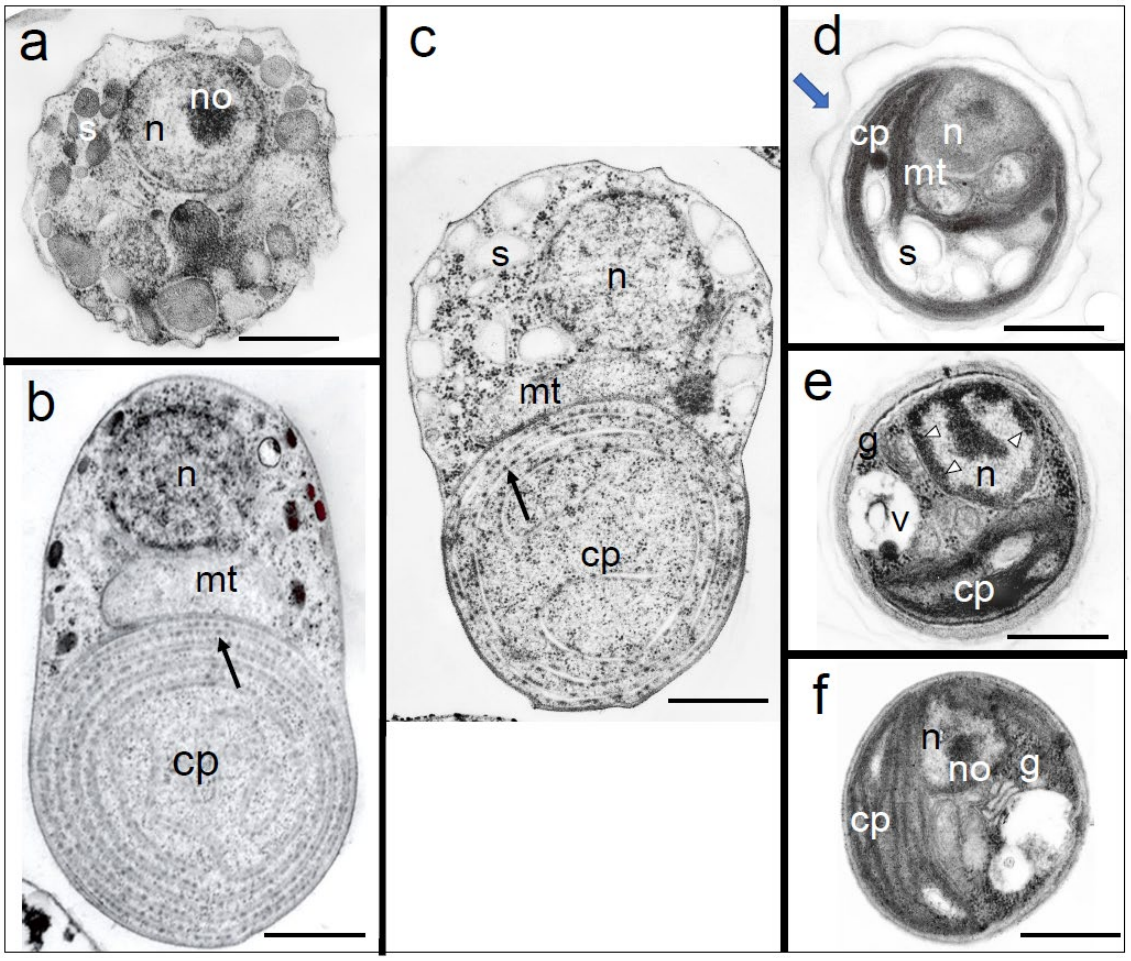

Supplementary Figure 2 Transmission electron microscopy images. **a–c** Images of *C. merolae* cells. Black thin arrows indicate phycobilisomes. **d–f** Images of *M. hakoo* cells. The blue thick arrow indicates thick cell walls, whereas arrowheads indicate an electron-dense chromatin structure; no, n, v, mt, cp, s, and g represent the nucleolus, nucleus, vacuole, mitochondrion, chloroplast, starch aggregates, and Golgi apparatus respectively. Bars represent 0.5 µm.

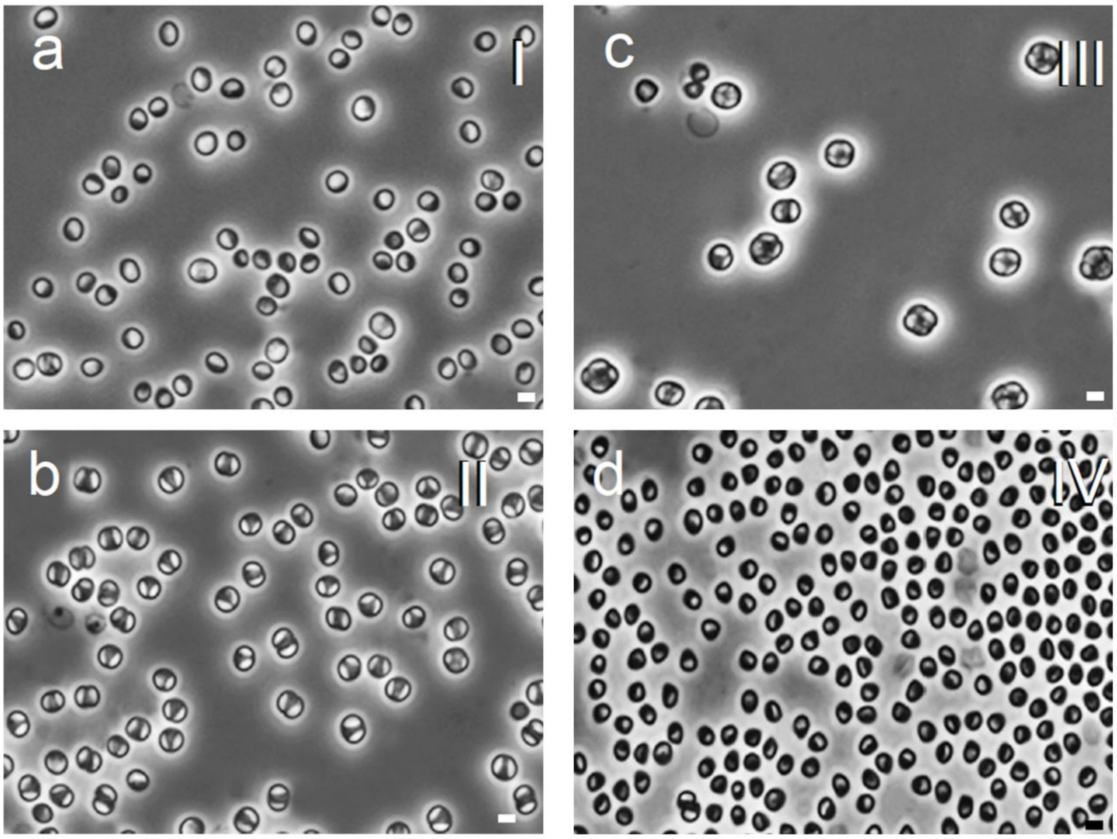

Supplementary Figure 3 Images of the *M. hakoo* synchronization culture. **a–d** Microscopy images of the *M. hakoo* synchronization cultured cells at specific time-points. Bars represent 1 µm.

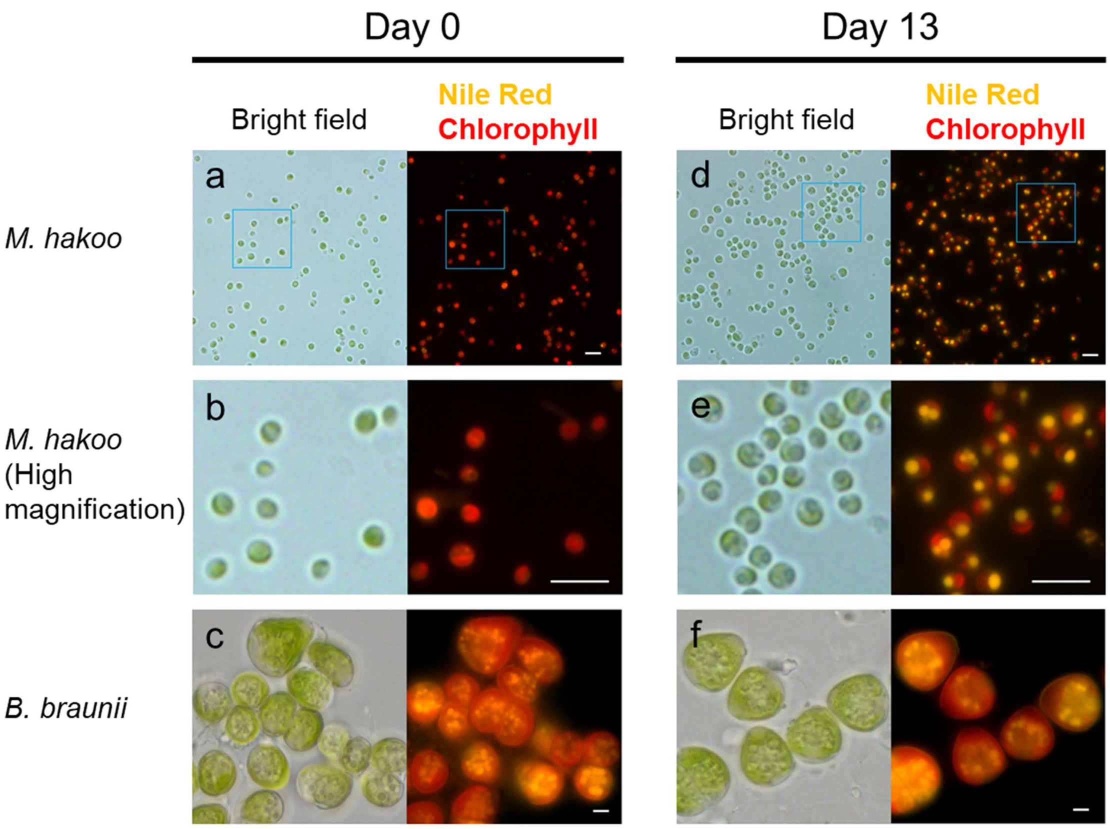

Supplementary Figure 4 Formation of lipid droplets in *M. hakoo* (**a** and **b, d** and **e**) and *B. braunii* (**c, f**). Both *M. hakoo* and *B. braunii* were cultured in nitrogen-depleted medium for 13 days and stained with Nile Red. The left panels present the bright-field images, whereas the right panels present the fluorescence images of the chloroplast (red) and lipid droplets (yellow). **a–c** and **d–f** Images from days 0 and 13, respectively. **b** and **e** Enlarged images of the samples presented in panels **a** and **d**, respectively. Scale bars indicate 1 μm.

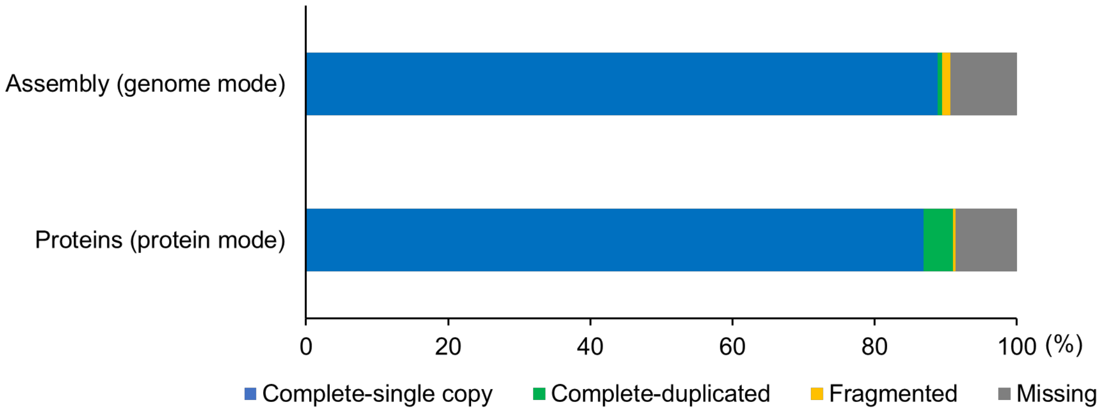

Supplementary Figure 5 BUSCO analysis of the *M. hakoo* genome.

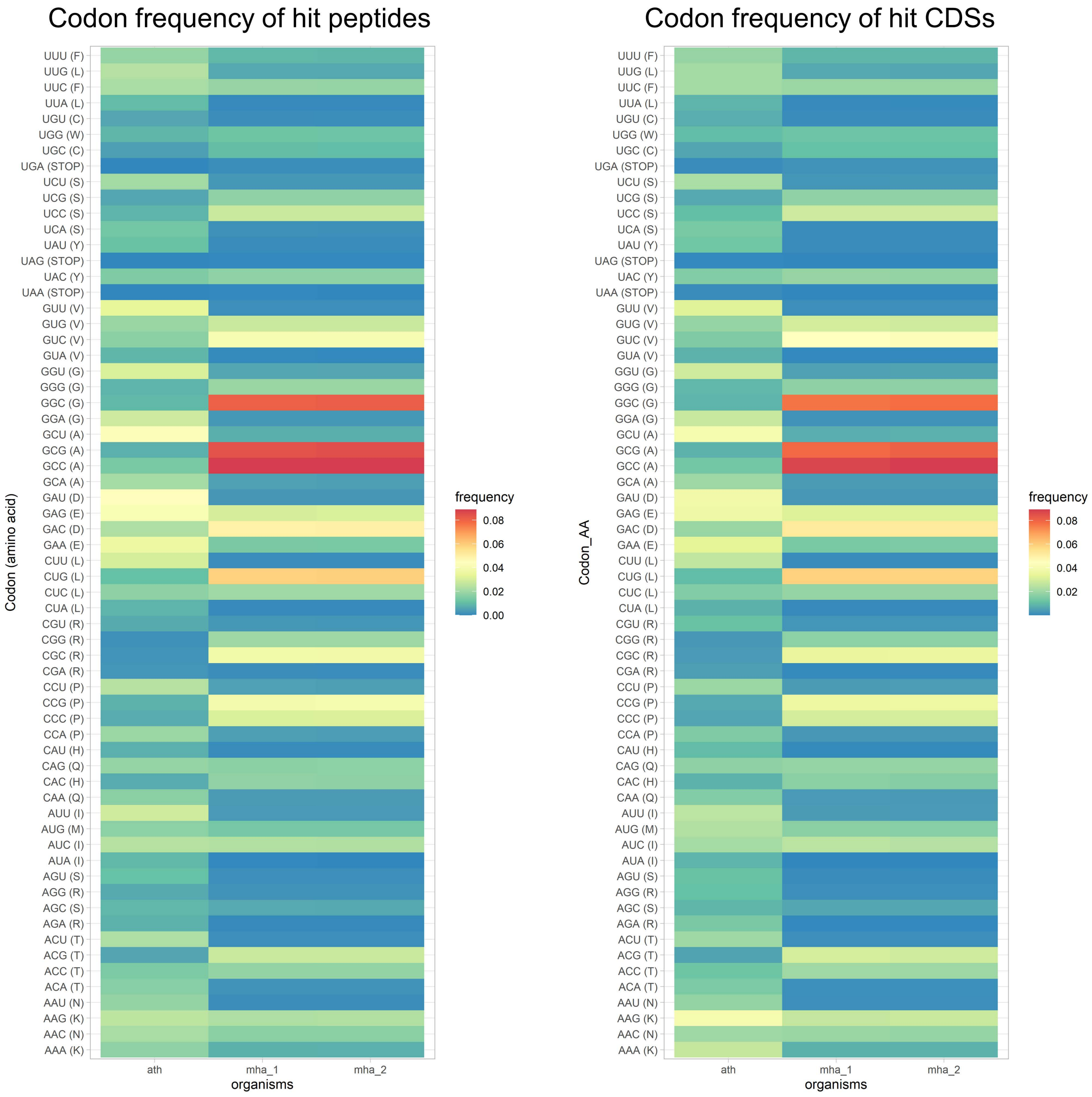

Supplementary Figure 6 Heat maps of the frequency of codons for the detected peptides and the complete coding sequences. The sample names mha and ath represent *M. hakoo* and *A. thaliana*, respectively. STOP represents a termination codon. The *M. hakoo* analyses were performed twice (i.e., technical replicates).

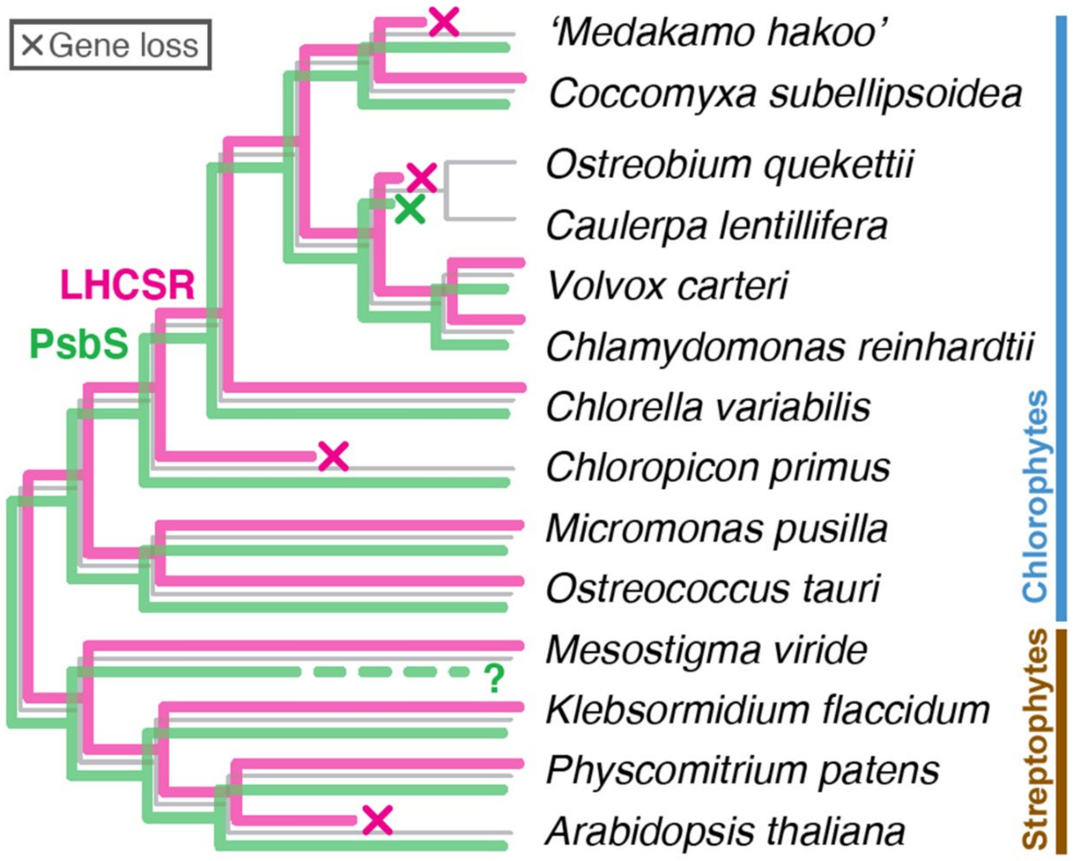

Supplementary Figure 7 Phylogenetic distribution of genes encoding LHCSR and PSBS proteins. Cross and question marks respectively indicate the absence of genes and the probable absence of genes (uncertainty due to the limitation of the gene model data).

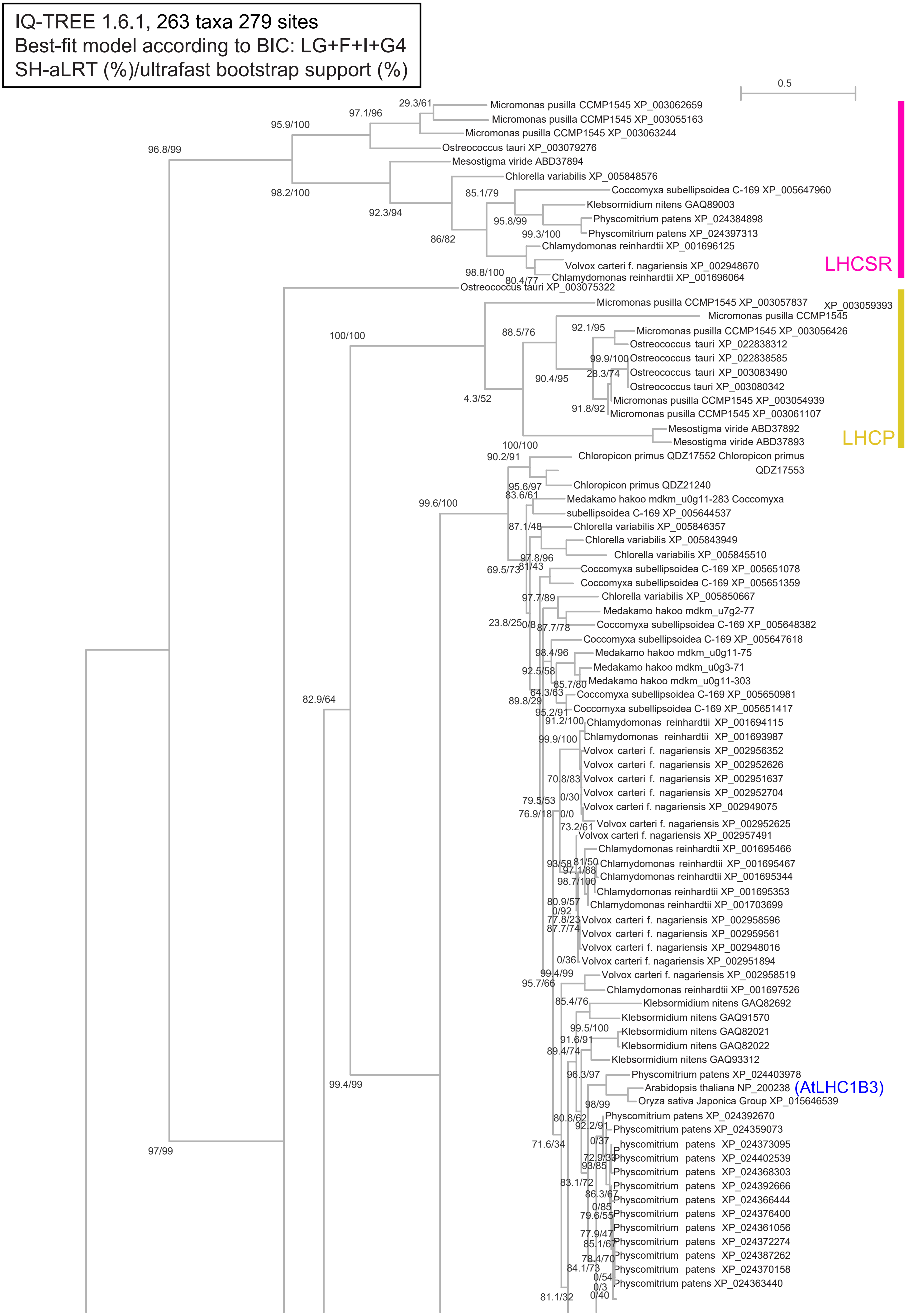
IQ-TREE 1.6.1, 263 taxa 279 sites
Best-fit model according to BIC: LG+F+I+G4
SH-aLRT (%)/ultrafast bootstrap support (%)
0.5
Micromonas pusilla CCMP1545 XP_003062659
Micromonas pusilla CCMP1545 XP_003055163
Micromonas pusilla CCMP1545 XP_003063244
Ostreococcus tauri XP_003079276
Mesostigma viride ABD37894
Chlorella variabilis XP_005848576
Coccomyxa subellipsoidea C-169 XP_005647960
Klebsormidium nitens GAQ89003
Physcomitrium patens XP_024384898
Physcomitrium patens XP_024397313
Chlamydomonas reinhardtii XP_001696125
Volvox carteri f. nagariensis XP_002948670
Chlamydomonas reinhardtii XP_001696064
LHCSR
Ostreococcus tauri XP_003075322
Micromonas pusilla CCMP1545 XP_003057837
Micromonas pusilla CCMP1545 XP_003059393
Micromonas pusilla CCMP1545 XP_003056426
Ostreococcus tauri XP_022838312
Ostreococcus tauri XP_022838585
Ostreococcus tauri XP_003083490
Ostreococcus tauri XP_003080342
Micromonas pusilla CCMP1545 XP_003054939
Micromonas pusilla CCMP1545 XP_003061107
Mesostigma viride ABD37892
Mesostigma viride ABD37893
LHCP
Chloropicon primus QDZ17552 Chloropicon primus QDZ17553
Chloropicon primus QDZ21240
Medakamo hakoo mdkm_u0g11-283 Coccomyxa subellipsoidea C-169 XP_005644537
Chlorella variabilis XP_005846357
Chlorella variabilis XP_005843949
Chlorella variabilis XP_005845510
Coccomyxa subellipsoidea C-169 XP_005651078
Coccomyxa subellipsoidea C-169 XP_005651359
Chlorella variabilis XP_005850667
Medakamo hakoo mdkm_u7g2-77
Coccomyxa subellipsoidea C-169 XP_005648382
Coccomyxa subellipsoidea C-169 XP_005647618
Medakamo hakoo mdkm_u0g11-75
Medakamo hakoo mdkm_u0g3-71
Medakamo hakoo mdkm_u0g11-303
Coccomyxa subellipsoidea C-169 XP_005650981
Coccomyxa subellipsoidea C-169 XP_005651417
Chlamydomonas reinhardtii XP_001694115
Chlamydomonas reinhardtii XP_001693987
Volvox carteri f. nagariensis XP_002956352
Volvox carteri f. nagariensis XP_002952626
Volvox carteri f. nagariensis XP_002951637
Volvox carteri f. nagariensis XP_002952704
Volvox carteri f. nagariensis XP_002949075
Volvox carteri f. nagariensis XP_002952625
Volvox carteri f. nagariensis XP_002957491
Chlamydomonas reinhardtii XP_001695466
Chlamydomonas reinhardtii XP_001695467
Chlamydomonas reinhardtii XP_001695344
Chlamydomonas reinhardtii XP_001695353
Chlamydomonas reinhardtii XP_001703699
Volvox carteri f. nagariensis XP_002958596
Volvox carteri f. nagariensis XP_002959561
Volvox carteri f. nagariensis XP_002948016
Volvox carteri f. nagariensis XP_002951894
Volvox carteri f. nagariensis XP_002958519
Chlamydomonas reinhardtii XP_001697526
Klebsormidium nitens GAQ82692
Klebsormidium nitens GAQ91570
Klebsormidium nitens GAQ82021
Klebsormidium nitens GAQ82022
Klebsormidium nitens GAQ93312
Physcomitrium patens XP_024403978
Arabidopsis thaliana NP_200238 (AtLHC1B3)
Oryza sativa Japonica Group XP_015646539
Physcomitrium patens XP_024392670
Physcomitrium patens XP_024359073
Physcomitrium patens XP_024373095
Physcomitrium patens XP_024402539
Physcomitrium patens XP_024368303
Physcomitrium patens XP_024392666
Physcomitrium patens XP_024366444
Physcomitrium patens XP_024376400
Physcomitrium patens XP_024361056
Physcomitrium patens XP_024372274
Physcomitrium patens XP_024387262
Physcomitrium patens XP_024370158
Physcomitrium patens XP_024363440

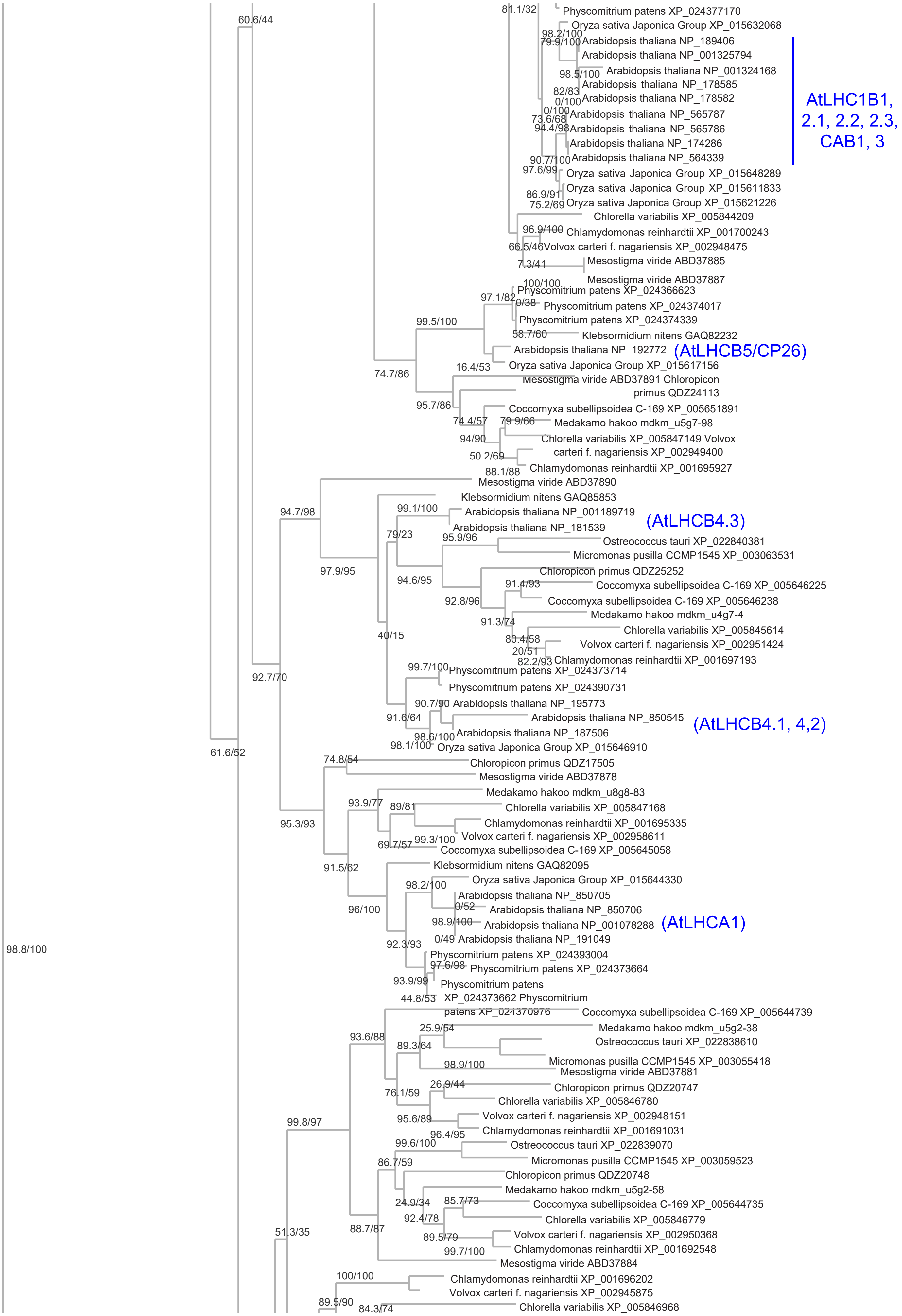
Physcomitrium patens XP_024377170
Oryza sativa Japonica Group XP_015632068
Arabidopsis thaliana NP_189406
Arabidopsis thaliana NP_001325794
Arabidopsis thaliana NP_001324168
Arabidopsis thaliana NP_178585
Arabidopsis thaliana NP_178582
Arabidopsis thaliana NP_565787
Arabidopsis thaliana NP_565786
Arabidopsis thaliana NP_174286
Arabidopsis thaliana NP_564339
Oryza sativa Japonica Group XP_015648289
Oryza sativa Japonica Group XP_015611833
Oryza sativa Japonica Group XP_015621226
Chlorella variabilis XP_005844209
Chlamydomonas reinhardtii XP_001700243
Volvox carteri f. nagariensis XP_002948475
Mesostigma viride ABD37885
Mesostigma viride ABD37887
AtLHC1B1, 2.1, 2.2, 2.3, CAB1, 3
Physcomitrium patens XP_024366623
Physcomitrium patens XP_024374017
Physcomitrium patens XP_024374339
Klebsormidium nitens GAQ82232
Arabidopsis thaliana NP_192772
(AtLHCB5/CP26)
Oryza sativa Japonica Group XP_015617156
Mesostigma viride ABD37891 Chloropicon primus QDZ24113
Coccomyxa subellipsoidea C-169 XP_005651891
Medakamo hakoo mdkm_u5g7-98
Chlorella variabilis XP_005847149 Volvox carteri f. nagariensis XP_002949400
Chlamydomonas reinhardtii XP_001695927
Mesostigma viride ABD37890
Klebsormidium nitens GAQ85853
Arabidopsis thaliana NP_001189719
Arabidopsis thaliana NP_181539
(AtLHCB4.3)
Ostreococcus tauri XP_022840381
Micromonas pusilla CCMP1545 XP_003063531
Chloropicon primus QDZ25252
Coccomyxa subellipsoidea C-169 XP_005646225
Coccomyxa subellipsoidea C-169 XP_005646238
Medakamo hakoo mdkm_u4g7-4
Chlorella variabilis XP_005845614
Volvox carteri f. nagariensis XP_002951424
Chlamydomonas reinhardtii XP_001697193
Physcomitrium patens XP_024373714
Physcomitrium patens XP_024390731
Arabidopsis thaliana NP_195773
Arabidopsis thaliana NP_850545
Arabidopsis thaliana NP_187506
(AtLHCB4.1, 4,2)
Oryza sativa Japonica Group XP_015646910
Chloropicon primus QDZ17505
Mesostigma viride ABD37878
Medakamo hakoo mdkm_u8g8-83
Chlorella variabilis XP_005847168
Chlamydomonas reinhardtii XP_001695335
Volvox carteri f. nagariensis XP_002958611
Coccomyxa subellipsoidea C-169 XP_005645058
Klebsormidium nitens GAQ82095
Oryza sativa Japonica Group XP_015644330
Arabidopsis thaliana NP_850705
Arabidopsis thaliana NP_850706
Arabidopsis thaliana NP_001078288
(AtLHCA1)
Arabidopsis thaliana NP_191049
Physcomitrium patens XP_024393004
Physcomitrium patens XP_024373664
Physcomitrium patens XP_024373662 Physcomitrium patens XP_024370976
Coccomyxa subellipsoidea C-169 XP_005644739
Medakamo hakoo mdkm_u5g2-38
Ostreococcus tauri XP_022838610
Micromonas pusilla CCMP1545 XP_003055418
Mesostigma viride ABD37881
Chloropicon primus QDZ20747
Chlorella variabilis XP_005846780
Volvox carteri f. nagariensis XP_002948151
Chlamydomonas reinhardtii XP_001691031
Ostreococcus tauri XP_022839070
Micromonas pusilla CCMP1545 XP_003059523
Chloropicon primus QDZ20748
Medakamo hakoo mdkm_u5g2-58
Coccomyxa subellipsoidea C-169 XP_005644735
Chlorella variabilis XP_005846779
Volvox carteri f. nagariensis XP_002950368
Chlamydomonas reinhardtii XP_001692548
Mesostigma viride ABD37884
Chlamydomonas reinhardtii XP_001696202
Volvox carteri f. nagariensis XP_002945875
Chlorella variabilis XP_005846968
98.8/100
61.6/52
60.6/44
92.7/70
94.7/98
97.9/95
95.3/93
91.5/62
99.8/97
51.3/35
74.7/86
99.5/100
95.7/86

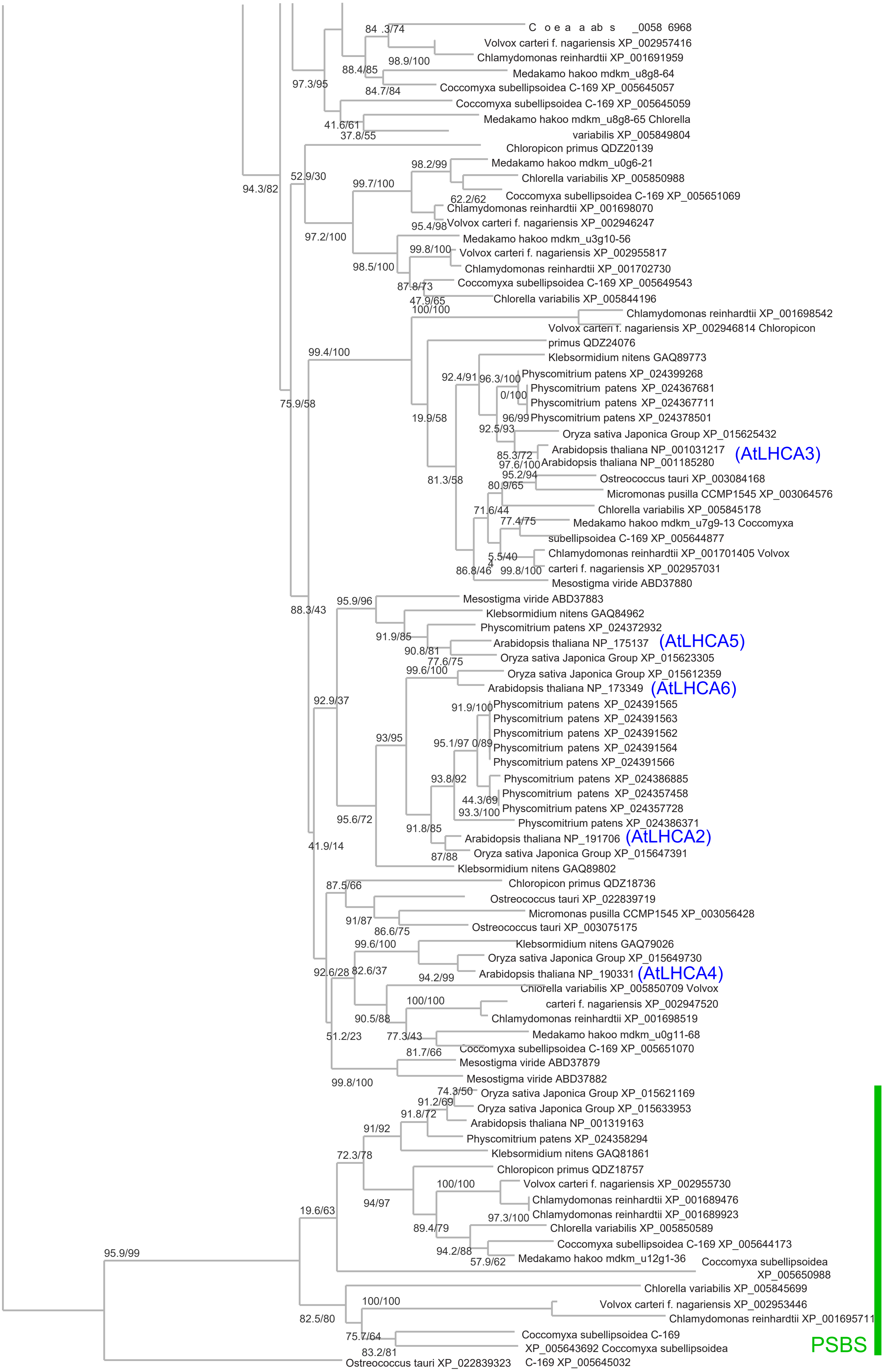

Supplementary Figure 8 Maximum-likelihood tree of green plant LHC-like proteins.

Supplementary Table 1 Information regarding the linear contigs in the *M. hakoo*

Chromosome No.	Length (bp)	GC content (%)	Contig No.
1	1,473,234	73.35	unitig_0
2	1,350,323	73.10	unitig_1
3	1,283,613	72.25	unitig_4
4	12,83,262	73.11	unitig_5
5	1,270,921	73.10	unitig_2
6	1,246,908	73.35	unitig_3
7	1,185,433	73.47	unitig_6
8	1,001,958	73.47	unitig_7
9	925,182	72.68	unitig_8
10	861,458	72.82	unitig_9
11	828,616	73.14	unitig_10
12	720,896	73.47	unitig_11
13	673,567	73.07	unitig_12
14	632,133	73.25	unitig_14
15	554,661	71.33	unitig_13
16	346,067	74.32	unitig_15

Supplementary Table 2 Predicted tRNAs in the *M. hakoo* genome.

tRNA	Amount of sequences
Ala	12
Arg	27
Asn	7
Asp	13
Cys	16
Gln	3
Glu	3
Gly	12
His	3
Ile	3
Leu	21
Lys	2
Met	5
Phe	10
Pro	9
Ser	40
Thr	9
Trp	1
Tyr	4
Val	3

Supplementary Table 3 G+C contents in the coding sequences and complete genome sequence of *M. hakoo*.

	GC bases	Total bases	GC (%)
**Coding region**	8,962,519	11,828,410	75.8
**Total genome**	11,427,589	15,638,232	73.1

Supplementary Table 4 Comparison of condensin- and cohesin-related genes between *C. merolae* and *M. hakoo*. The KEGG Orthology analysis was performed using the GhostKOALA[3] online program (https://www.kegg.jp/ghostkoala/). A BLASTP analysis was performed using each gene in the *M. hakoo* genome as a query to screen the *C. merolae* genome. The number of *M. hakoo* genes that matched a *C. merolae* gene was recorded.

**Subunit name**	**ID**	**Gene name**	**KOnumber**	**Number of genes annotated as each KOnumber in the genome of *M. hakoo***	**Number of BLAST hit genes in *M. hakoo* genome (Each genes of *C. merolae* were used as a query)**
Condensin	CMG189C	SMC2; structural maintenance of chromosome 2	K06674	1	1
	CME029C	SMC4; structural maintenance of chromosome 4	K06675	1	1
I specific	CMF069C	BRRN1; condensin complex subunit 2	K06676	1	1
	CMR484C	YCS4; condensin complex subunit 1	K06677	1	2
	CMS422C	YCG1; condensin complex subunit 3	K06678	1	1
II specific	CMI207C	NCAPH2; condensin-2 complex subunit H2	K11490	1	1
	CMQ236C	NCAPD3; condensin-2 complex subunit D3	K11491	1	1
	CMA089C	NCAPG2; condensin-2 complex subunit G2	K11492	1	1
Cohesin	CMI192C	SMC1; structural maintenance of chromosome 1	K06636	1	1
	CML027C	SMC3; structural maintenance of chromosome 3 (chondroitin sulfate proteoglycan 6)	K06669	1	1
	CML311C	SCC1; cohesin complex subunit SCC1	K06670	1	1
	CMO331C	STAG1_2; cohesin complex subunit SA-1/2 SCC3	K06671	0	1
	CMQ352C	PDS5; sister chromatid cohesion protein PDS5	K11267	1	0
SMC5/6	CMH246C	SMC5; structural maintenance of chromosomes protein 5	K22803	1	2
	CMA066C	SMC6; structural maintenance of chromosomes protein 6	K22804	1	2
	CMP167C	NSMCE2; E3 SUMO-protein ligase NSE2 [EC:2.3.2.-]	K22756	0	0
	CMQ181C	NSMCE4; non-structural maintenance of chromosomes element 4	K22825	1	1
	CMP167C	NSMCE2; E3 SUMO-protein ligase NSE2 [EC:2.3.2.-]	K22756	0	0

Supplementary Table 5 Relationship between the conservation of RNAi-related genes and genome size. The conservation of RNAi-related genes was assessed according to the genes annotated by the KEGG Orthology analysis, which was performed using the GhostKOALA[3] online program (https://www.kegg.jp/ghostkoala/).

Classification	Organisms	Genome size (bp)	Protein coding genes	K11592_Dicer	K11593_Argonaute
Eudicots	*Arabidopsis thaliana*	1.20E+08	27,636	4	10
Eudicots	*Glycine max*	9.79E+08	46,993	8	20
Monocots	*Oryza sativa*	3.74E+08	28,073	6	18
Ferns	*Selaginella moellendorffii*	2.12E+08	34,724	7	12
Mosses	*Physcomitrella patens*	4.72E+08	20,454	4	7
Basal Magnoliophyta	*Amborella trichopoda*	7.06E+08	17,106	4	10
Green algae	*Chlamydomonas reinhardtii*	1.20E+08	14,415	1	2
Green algae	*Volvox carteri f. nagariensis*	1.38E+08	14,434	0	2
Green algae	*Monoraphidium neglectum*	69,711,829	16,734	0	1
Green algae	*Ostreococcus lucimarinus CCE9901*	13,204,888	7,603	0	0
Green algae	*Ostreococcus tauri*	13,032,761	7,765	0	0
Green algae	*Bathycoccus prasinos*	15,074,320	7,892	0	0
Green algae	*Micromonas commoda*	21,109,336	10,049	0	1
Green algae	*Micromonas pusilla CCMP1545*	21,958,260	10,238	0	0
Green algae	*Coccomyxa subellipsoidea C-169*	48,826,616	9,945	0	1
Green algae	*Chlorella variabilis*	46,159,512	9,892	1	1
Green algae	*Auxenochlorella protothecoides*	22,924,637	7,127	1	1
Green algae	*Medakamo hakoo*	15,811,321	6,399	0	0
Red algae	*Cyanidioschyzon merolae strain 10D*	16,546,747	5,010	0	0
Red algae	*Galdieria sulphuraria*	13,712,004	6,823	0	0
Red algae	*Chondrus crispus*	1.05E+08	9,836	2	3

Supplementary Table 6 Autophagy-related genes in *M. hakoo.* The conservation of autophagy-related genes was predicted according to the KEGG Orthology method and the BLAST-based method used to generate the data presented in Supplementary Tables 4–6.

*Medakamo*_KO_annotation	*Medakamo*_blast_annotation	Locus Identifier	Primary Gene Symbol
1	1	AT5G61500	(ATG3)
1	0	AT3G59950	(ATG4B)
1	0	AT2G05630	(ATG8D)
1	0	AT3G57090	(BIGYIN)
0	1	AT5G43560	(MUSE14)
1	2	AT3G08850	(RAPTOR1)
1	0	AT5G16280	(TRAPPC8)
1	1	AT5G53000	2A PHOSPHATASE ASSOCIATED PROTEIN OF 46 KD (TAP46)
0	1	AT2G34690	ACCELERATED CELL DEATH 11 (ACD11)
1	1	AT3G07525	AUTOPHAGY 10 (ATG10)
1	1	AT1G54210	AUTOPHAGY 12 A (ATG12A)
1	0	AT3G13970	AUTOPHAGY 12 B (APG12B)
1	0	AT3G18770	AUTOPHAGY 13B (ATG13B)
1	1	AT5G50230	AUTOPHAGY 16 (ATG16)
0	1	AT3G19190	AUTOPHAGY 2 (ATG2)
1	1	AT2G44140	AUTOPHAGY 4A (ATG4A)
1	1	AT5G17290	AUTOPHAGY 5 (APG5)
1	0	AT3G61710	AUTOPHAGY 6 (ATG6)
2	2	AT5G45900	AUTOPHAGY 7 (APG7)
1	0	AT4G21980	AUTOPHAGY 8A (APG8A)
1	0	AT4G04620	AUTOPHAGY 8B (ATG8B)
1	0	AT1G62040	AUTOPHAGY 8C (ATG8C)
1	0	AT2G45170	AUTOPHAGY 8E (ATG8E)
1	1	AT4G16520	AUTOPHAGY 8F (ATG8F)
1	0	AT3G60640	AUTOPHAGY 8G (ATG8G)
1	0	AT3G15580	AUTOPHAGY 8H (APG8H)
1	0	AT3G06420	AUTOPHAGY 8H (ATG8H)
1	1	AT2G31260	AUTOPHAGY 9 (APG9)
1	1	AT5G66930	AUTOPHAGY-RELATED 101 (ATG101)
1	1	AT4G30790	AUTOPHAGY-RELATED 11 (ATG11)
1	0	AT3G49590	AUTOPHAGY-RELATED 13A (ATG13a)
1	1	AT3G53930	AUTOPHAGY-RELATED PROTEIN 1B (ATG1B)
1	0	AT2G37840	AUTOPHAGY-RELATED PROTEIN 1C (ATG1C)
0	1	AT4G36630	EMBRYO DEFECTIVE 2754 (EMB2754)
1	1	AT5G12390	FISSION 1B (FIS1B)
0	1	AT1G20110	FYVE-DOMAIN PROTEIN 1 (FYVE1)
1	1	AT4G30510	HOMOLOG OF YEAST AUTOPHAGY 18 (ATG18) B (ATG18B)
1	1	AT3G18140	LETHAL WITH SEC THIRTEEN 8-1 (LST8-1)
1	0	AT5G47480	MAIGO 5 (MAG5)
0	1	AT3G54360	NO CATALASE ACTIVITY 1 (NCA1)
1	0	AT1G22740	RAB GTPASE HOMOLOG G3B (RABG3B)
1	0	AT5G01770	RAPTOR2 (RAPTOR2)
1	0	AT2G39780	RIBONUCLEASE 2 (RNS2)
1	1	AT3G01090	SNF1 KINASE HOMOLOG 10 (KIN10)
1	4	AT1G50030	TARGET OF RAPAMYCIN (TOR)
1	1	AT4G02030	UNHINGED (UNH)
1	2	AT4G29380	VACUOLAR PROTEIN SORTING 15 (VPS15)
0	1	AT1G22860	VACUOLAR PROTEIN SORTING 3 (VPS3)
1	1	AT1G60490	VACUOLAR PROTEIN SORTING 34 (VPS34)
1	0	AT1G17730	VACUOLAR PROTEIN SORTING 46.1 (VPS46.1)

Supplementary Table 7 Effect size of Brunner Munzel testin Figure 5c (P(X<Y)+0.5*P(X=Y))

X	Y	Effect size
CE	AS	0.506272
CE	*M. hakoo*-specific	0.383982
AS	CE	0.493728
AS	*M. hakoo*-specific	0.380155
M. hakoo-specific	CE	0.616018
M. hakoo-specific	AS	0.619845

Supplementary Table 8 Composition of the growth media used for the lipid formation assay.

Components	N- media (mM)	Normal media (mM)
$CaCl_2$	1	-
$CaCl_2 \cdot 2H_2O$	-	2.993
$MgSO_4 \cdot 7H_2O$	4	1.502
KH_2PO_4	8	1.249
KNO_3	-	18.791
KI	-	0.005
NH_4NO_3	-	20.612
Na_2SO_4	40	-
H_3BO_3	0.1844	0.100
$CuCl_2$	0.0013	-
$CuSO_4$	-	0.000157
$CoCl_2 \cdot 6H_2O$	0.0007	0.000105
$MnCl_2 \cdot 4H_2O$	0.0364	-
$MnSO_4 \cdot 4H_2O$	-	0.100
$Na_2MoO_4 \cdot 2H_2O$	0.0064	0.001
$FeCl_3$	0.0986	-
$FeSO_4 \cdot 7H_2O$	-	0.100
EDTA-2Na	-	0.100
EDTA-2NA$\cdot 2H_2O$	0.0827	-
$ZnCl_2$	0.0031	-
$ZnSO_4 \cdot 7H_2O$	-	0.030

Supplementary Note 1 Formal taxonomic treatment

Medakamo T. Kuroiwa gen. nov.

Registration: http://phycobank.org/103506

Description

Cells solitary, symmetrically spherical or ellipsoidal in shape, with a nucleus, a mitochondrion, and a chloroplast without pyrenoids, enclosed tightly by a smooth cell wall, measuring approximately 1–3 µm in diameter. During asexual reproduction, daughter cells are formed within a parental cell wall. Sexual reproduction is unknown. Phylogenetic position differs from that of other genera according to *rbcL* sequences.

Type species designated here:

Medakamo hakoo T. Kuroiwa sp. nov.

Etymology

The Japanese noun "*Medaka-mo*" refers to an alga living with killifish. Treated as a feminine noun.

Medakamo hakoo T. Kuroiwa sp. nov.

Registration: http://phycobank.org/103507

Description

Cells solitary, symmetrically ellipsoidal or nearly spherical in shape, with a nucleus, a mitochondrion, and a chloroplast, enclosed tightly by smooth cell walls, measuring approximately 1 µm in diameter. The nucleus is positioned in the center of cells. The chloroplast is cup- or urn-shaped, occupying most of the cell periphery, containing starch granules but lacking pyrenoids. During asexual reproduction, two successive equal cell divisions form four daughter cells of identical size within a parental cell wall. Sexual reproduction is unknown.

Holotype designated here: Cryopreserved culture of *M-hakoo* 311 has been deposited in the Microbial Culture Collection at the National Institute for Environmental Studies (MCC-NIES), Tsukuba, Ibaraki, Japan[2] as "NIES-50025".

Nuclear and organellar genome sequences were deposited in DDBJ/NCBI/GenBank with Accession No. CP089450–CP089465 (nuclear genome), LC604816 (plastid genome), and LC604817 (mitochondrial genome).

Type locality: Kagurazaka, Shinjuku-ku, Tokyo, Japan (35.70269509974511 N, 139.73750777817125 E); sampling date: March 11, 2014.

Habitat

Artificial aquarium, freshwater.
Authentic culture
M-hakoo 311. This culture is maintained as NIES-4000 in MCC-NIES. The cryopreserved holotype culture of NIES-4000 is designated as "NIES-50025" and has been deposited in MCC-NIES.
Etymology
The specific epithet "*hakoo*" refers to the nickname given to Dr. Haruko Kuroiwa, known for loving plants and algae. Treated as a feminine noun.

Medakamo limnetica (Pröschold & Darienko) T. Kuroiwa comb. nov.
Basionym: *Choricystis limnetica* Pröschold & Darienko 2020: 187, Fig. 1e.
Registration: http://phycobank.org/103508

Remarks: Among freshwater coccoid green algae[3], *Medakamo hakoo* is similar to *Choricystis coccoides* (Rodhe & Skuja) Fott, with both species having extremely small unicells (approximately 0.5–1 µm in diameter). However, these two species differ in cell and chloroplast shapes. Specifically, *M. hakoo* cells are symmetrically spherical or ellipsoidal in shape, with a cup- or urn-shaped chloroplast. In contrast, *C. coccoides* cells are elongate or lunate and asymmetrical in shape, with a dorsal side and a flattened or concave ventral side, and contain a parietal chloroplast occupying the dorsal side and a nucleus on the ventral side[4]. Other *Choricystis* species also have elongate and asymmetrical cells with dorsal and ventral sides[3,5], with the exception of *Choricystis limnetica* Pröschold & Darienko[6]. According to Pröschold and Darienko[6], the spherical cells of *C. limnetica* differentiate it from other *Choricystis* species. Furthermore, *M. hakoo* and *C. limnetica* form a monophyletic group that is phylogenetically separate from a large clade containing numerous genotypes classifiable as *C. parasitica* (K. Brandt) Pröschold & Darienko [= *C. minor* (Skuja) Fott] and *C. krienitzii* Pröschold & Darienko on the basis of phylogenetic taxonomic concepts[6] (Fig. 2b). Thus, a new genus is proposed here to accommodate *M. hakoo* and *C. limnetica*. *Medakamo* is monophyletic and distinguished from *Choricystis* in having spheroidal or symmetrical unicells and a different phylogenetic position.

Supplementary Data 1 Functional annotation of predicted CDSs with eggNOG-mapper.

Supplementary Data 2 Functional annotation of predicted CDSs with GhostKOALA.

Supplementary Data 3 Results of the *M. hakoo* proteome analysis.

Supplementary Data 4 Matrix of orthogroup composition of each algal genome.

Supplementary Data 5 AC, CE and AS gene sets.

Supplementary Data 6 Source data of Fig 1h, 3b, 3e, 4b-c, 5b-d.

Supplementary References

1. Kanehisa, M., Sato, Y. & Morishima, K. BlastKOALA and GhostKOALA: KEGG tools for functional characterization of genome and metagenome sequences. *J. Mol. Biol.* **428**, 726–731 (2016).
2. Kawachi, M., Ishimoto, M., Mori, F., Yumoto, K., Sato, M., Noël, M.-H. MCC-NIES list of strains, 9th edition, microbial culture collection at National Institute for Environmental Studies, Tsukuba, Japan. https://mcc.nies.go.jp/download/list9th_e.pdf (2013).
3. Komarek, J. Chlorophyceae (Grunalgen), Ordnung: Chlorococcales. *Das Phytoplankton des Susswassers. Systematik und Biologie* **7**, (1983).
4. Skuja, H. Taxonomie des Phytoplanktons einiger Seen in Uppland, Schweden. *Symb. Bot. Ups.* **9**, 1–399 (1948).
5. Krienitz, L., Huss, V. A. R. & Hümmer, C. Picoplanktonic Choricystis species (Chlorococcales, Chlorophyta) and problems surrounding the morphologically similar 'Nannochloris-like algae.' *Phycologia* **35**, 332–341 (1996).
6. Pröschold, T. & Darienko, T. *Choricystis* and *Lewiniosphaera* gen. nov. (Trebouxiophyceae Chlorophyta), two different green algal endosymbionts in freshwater sponges. *Symbiosis* **82**, 175–188 (2020).
