# Supplementary material for: Genomic analysis of an ultrasmall freshwater green alga, Medakamo hakoo
Source: Commun Biol. 2023 Jan 23;6:89. doi: 10.1038/s42003-022-04367-9 (PMC9871001; doi:10.1038/s42003-022-04367-9)
Supplement: Supplementary file 3 — Description of Additional Supplementary Data [file 42003_2022_4367_MOESM3_ESM.docx]

**Description of Additional Supplementary Files**

**File name:** Supplementary Data 1

**Description:** Functional annotation of predicted CDSs with eggNOGmapper.

**File name:** Supplementary Data 2

**Description:** Functional annotation of predicted CDSs with GhostKOALA.

**File name:** Supplementary Data 3

**Description:** Results of the M. hakoo proteome analysis.

**File name:** Supplementary Data 4

**Description:** Matrix of orthogroup composition of each algal genome.

**File name:** Supplementary Data 5

**Description:** AC, CE and AS gene sets.

**File name:** Supplementary Data 6

**Description:** Source data of Fig 1h, 3b, 3e, 4b-c, 5b-d.
